# Possible relationship between mitochondrial changes and oxidative stress under low dose-rate irradiation

**DOI:** 10.1080/13510002.2021.1971363

**Published:** 2021-08-26

**Authors:** Qingmei Meng, Elena Karamfilova Zaharieva, Megumi Sasatani, Junya Kobayashi

**Affiliations:** a Department of Interdisciplinary Environment, Graduate School of Human and Environmental Sciences, Kyoto University, Yoshidanihonmatsucho, Sakyo-ku, Kyoto, Japan; b Department of Experimental Oncology, Research Institute for Radiation Biology and Medicine (RIRBM), Hiroshima University, Hiroshima, Japan; c Department of Radiological Sciences, School of Health Sciences at Narita, International University of Health and Welfare, Narita, Japan

**Keywords:** Low dose-rate irradiation, oxidative stress, mitochondria, mitophagy, ROS, PINK1, DNA damage, genomic instability, ATM

## Abstract

**Objectives:** High dose-rate ionizing radiation (IR) causes severe DSB damage, as well as reactive oxygen species (ROS) accumulation and oxidative stress. However, it is unknown what biological processes are affected by low dose-rate IR; therefore, the molecular relationships between mitochondria changes and oxidative stress in human normal cells was investigated after low dose-rate IR.

**Methods:** We compared several cellular response between high and low dose-rate irradiation using cell survival assay, ROS/RNS assay, immunofluorescence and western blot analysis.

**Results:** Reduced DSB damage and increased levels of ROS, with subsequent oxidative stress responses, were observed in normal cells after low dose-rate IR. Low dose-rate IR caused several mitochondrial changes, including morphology mass, and mitochondrial membrane potential, suggesting that mitochondrial damage was caused. Although damaged mitochondria were removed by mitophagy to stop ROS leakage, the mitophagy-regulatory factor, PINK1, was reduced following low dose-rate IR. Although mitochondrial dynamics (fission/fusion events) are important for the proper mitophagy process, some mitochondrial fusion factors decreased following low dose-rate IR.

**Discussion:** The dysfunction of mitophagy pathway under low dose-rate IR increased ROS and the subsequent activation of the oxidative stress response.

## Introduction

Various biological effects occur when cells are exposed to ionizing radiation (IR). Among these biological effects, DNA damage, especially DNA double-strand breaks (DSBs), has the most severe impact on cells, as accumulation of unrepaired DSB damage triggers genome instability, a hallmark of tumorigenesis [1]. Hence, to maintain genome integrity, mammalian cells have developed intricate DNA damage responses (DDRs), such as cell cycle checkpoints and DNA repair mechanisms [2]. Exposure of cells to IR can also frequently generates ROS including free radicals such as superoxide (O_2__·_^–^) in addition to hydrogen peroxide (H_2_O_2_) through the radiolysis of water. Such ROS could react with biomolecules immediately (less than μS timescale) in cells, such as nucleic acids, proteins, and lipids, leading to oxidative DNA damage, irreversible modification of proteins and lipids, oxidative injury of several organelles, and abnormal redox homeostasis [3]. Such accumulation of damaged biomolecules induced by ROS elicits several biological responses, such as inflammation, mutation, carcinogenesis, and cell death [3, 4].

High dose-rate IR causes severe DSB damage and abnormal accumulation of endogenous reactive oxygen species (ROS) and oxidative stress in mammalian cells; exposure of mammalian cells to 1 gray (Gy) of high dose-rate γ-rays is estimated to generate approximately 50 DSBs into genomic DNA per nucleus [1]. However, exposure of MCF-7 cells to 2 or 4 Gy of X-rays causes transient ROS accumulation until 24 h, which is reduced to basal levels within 72 h after radiation [5]. Irradiation of U937 cells by γ-rays also elevates ROS levels until 12 h post-irradiation [6].

As the major ROS generator in cell, mitochondria produces superoxide during the process of oxidative process which could be converted to H_2_O_2_ by superoxide dismutase [7]. A variety of environmental stresses or agent treatments cause ROS accumulation, accompanied by various mitochondrial changes related to mitochondrial damage [8–10]. For example, heat stress disturbs the function and structure of complex I of the ETC, resulting in the reduction of ATP synthesis and subsequent ROS increase [8]. In addition, medication treatment, such as pyocyanin (an electron receptor), induces mitochondrial damage by reducing complex III activity and disrupting electron transport in the mitochondrial electron transfer chain (ETC), resulting in the reduction of ATP synthesis, mitochondrial membrane potential (MMP) as well as increased oxygen consumption and subsequent ROS increases [9, 10]. The mitochondrial changes such as ATP reduction are used as the indicator of mitochondrial damage. Such damaged mitochondria lead to ROS leakage to the cytoplasm, which reacts with important biomolecules, leading to oxidative stress and genomic instability [11, 12].

To maintain mitochondrial homeostasis and repress excess ROS accumulation in a cell, damaged mitochondria are usually recognized and removed by a process known as mitophagy, a mitochondria-specific autophagy system [13]. When mitochondrial damage occurs, PTEN-induced kinase 1 (PINK1) is stabilized on the outer mitochondrial membrane, promoting Parkin recruitment to the mitochondria membrane. Parkin ubiquitinates several mitochondria-associated proteins that are recognized by p62. Then p62 binds to light chain 3 (LC3) to regulate the mitochondrial elimination [14, 15]. Mitochondrial dynamics plays an important role in mitophagy, since mitochondria can isolate the impaired fraction to small individual mitochondria through fission events for elimination by mitophagy, whereas the healthy fraction will be brought back to the interconnected mitochondria network through fusion events [16, 17].

Mitochondrial changes induced by high dose-rate IR have been reported in many studies. Wang et al. have found a 3.2-fold increase in the mitochondrial mass in HepG2 cells 24 h after exposure to 5 Gy of X-rays [18]. Yoshida et al. have shown that human osteosarcoma cells undergo a 20% reduction in ATP content 1 h after irradiation with 8 Gy of γ-rays, followed by a full recovery approximately 3 h later [19]. Furthermore, Kim et al. have reported that irradiation of U937 cells with 7 Gy of γ-rays causes both the accumulation of ROS and MMP loss starting from 10 h post-irradiation, reaching the first peak at 12 h post-irradiation, following a slight recovery at 14 h post-irradiation. Such fluctuations in ROS accumulation and MMP loss are observed in a tightly associated manner until 30 h post-irradiation [6]. It is likely that the transient increase in ROS levels upon high dose-rate IR may be caused by several mitochondrial changes, and a subsequent reduction of ROS may be associated with mitophagy, the elimination process of damaged mitochondria.

Although the amount of DSB damage is remarkably reduced under low dose or low dose-rate IR, there is little information on the biological effects of such irradiation. Nakamura et al. [20] have reported that irradiation of telomerase reverse transcriptase (TERT)-immortalized human fibroblasts with low dose-rate γ-rays (0.3 mGy/min) reduces micronuclei formation and mutation frequency of the hypoxanthine-guanine phosphoribosyltransferase (*HPRT*) gene and increases viability compared to high dose-rate X-ray (2 Gy/min) irradiation. Nakamura et al. [21] have also found that normal cells show a marked increase in cell viability and a remarkable reduction of micronuclei and γ-H2AX focus formation upon low dose-rate IR compared to cells receiving high dose-rate IR. However, irradiation of patient cells derived from ataxia-telangiectasia (AT) disorder, which is caused by mutations in the ataxia-telangiectasia mutated (*ATM*) gene, with low dose-rate IR shows no difference in cell viability and micronucleus formation compared to cells with high dose-rate IR; the number of γ-H2AX foci is reduced in a dose-rate-dependent manner. Moreover, elevated auto-phosphorylation of ATM kinase is observed even after exposure to low dose-rate IR in normal cells. It is well known that ATM kinase is activated in response to DSB damage and subsequently phosphorylates various proteins involved in DDRs, such as DNA repair mechanisms and cell cycle checkpoints [22]. Accumulating evidence has demonstrated that ATM is also activated by ROS and regulates a number of processes to promote restoration of redox homeostasis [23, 24]. Furthermore, micronuclei formation is induced by treatment with H_2_O_2_ [25]. Therefore, ATM kinase may repress micronuclei formation induced by low dose-rate IR and ROS, and hence the dysfunction of ATM in AT cells may cause micronuclei formation associated with ROS. Perhaps, low dose-rate IR may cause excess ROS accumulation, which may induce biological effects in irradiated cells.

Continuous excess accumulation of ROS causes oxidative stress and induces genomic instability, which contributes to an increased risk of carcinogenesis and neurodegeneration [26–28]. Therefore, the possibility of excess ROS accumulation under low dose-rate IR, the biological effects and molecular mechanisms in human normal cells are important. It has been reported that biological effects such as somatic mutations (*HPRT* mutation) and cell inactivation are dose rate dependent and exhibit a minimum at dose rates from 0.1 mGy/min to 10 mGy/min [29]. Also, studies had shown that low dose-rate IR could induce detrimental effects such as premature senescence and secretion of pro-inflammatory molecules when the total dose was higher than 2 Gy [30, 31]. Therefore, we use the low dose-rate IR of 1 mGy/min, which falls in the range of observed minimum, and compare with high dose-rate IR (0.9 Gy/min), in order to investigate the different biological effects of low dose-rate IR and high dose-rate IR at the same total dose of 3 Gy.

In this study, ROS generation was investigated, and the molecular relationship between ROS increase and mitochondrial homeostasis upon low dose-rate IR was clarified. Consequently, ROS accumulation and subsequent activation of the oxidative stress response was observed. Changes in the protein expression of several regulators of mitochondrial dynamics and their effects on mitophagy were also observed. Finally, the relationship among ROS increase, mitochondrial dynamics, and mitophagy under low dose-rate IR is discussed.

## Materials and methods

### Cell culture

HeLa and hTERT-immortalized human fibroblasts (48BR) were cultured in Dulbecco’s modified Eagle’s medium (DMEM; Sigma-Aldrich) supplemented with 10% fetal bovine serum (FBS; Invitrogen) and antibiotics [32].

### Gamma-ray irradiation and drug treatment

High dose-rate γ-ray irradiation was performed using a Gammacell 40 Exactor (Nordion Inc., Kanata, Canada). The radioisotope source was ^137^Cs (132.2 TBq) and the dose rate was 0.9 Gy/min. Low dose-rate *γ*-ray irradiation was performed using a low dose and low dose-rate irradiation system (Sangyo Kagaku Co., Ltd., Tokyo, Japan). The radioisotope source was ^137^Cs (1.85 TBq), and the dose rate was 1 mGy/min [20]. During low dose-rate IR, the cells were maintained at 37°C in a humidified 5% CO_2_ incubator (normal cell culture condition), while high dose-rate IR was carried out in the atmosphere.

Pyocyanin is a mitochondrial ROS inducer [33]. Pyocyanin (Sigma) treatment (10 µM) was performed for 2 h. Bafilomycin A (BA), a specific inhibitor of the vacuolar type H (+)-ATPase in cells, inhibits the fusion of autophagosomes with lysosomes by inhibiting the acidification of organelles containing this enzyme (such as lysosomes and endosomes) [34]. BA (Cell Signaling Technology) treatment (100 nM) was performed for 46 h, including irradiation time. KU55933 (Sigma) is an ATM-kinase inhibitor. Cells were treated with 10 μM KU55933 for 46 h, including irradiation time.

### Antibodies

The following antibodies were used in this study: mouse monoclonal anti-γH2A histone family X (H2AX) [#05-636] and rabbit polyclonal anti-H2B [#07-371] (Millipore Co.); rabbit polyclonal anti-phospho-KRAB-associated protein-1 (KAP1) [A300-767A] and mouse monoclonal anti-KAP1 [GTX49179] (Bethyl Laboratories Inc.); rabbit polyclonal anti-p53-binding protein 1 (53BP1, Thermo Science); rabbit polyclonal anti-phospho-AMP-activated protein kinase α (AMPKα) (Thr172) [#2535], anti-phospho-p38 mitogen-activated protein kinase (MAPK) (Thr180/Thr182) [#4511], and anti-LC3B [#2775] (Cell Signalling Technology); rabbit polyclonal anti-MFN1 [13798-1-AP], anti-MFN2 [12186-1-AP], anti-mitochondrial fission factor (MFF) [17090-1-AP], anti-translocase of outer mitochondrial membrane 20 (TOMM20) [11802-1-AP], anti-PARK2/Parkin [14060-1-AP], anti-p62/Sequestosome 1 [18420-1-AP], and mouse monoclonal anti-p38 MAPK [66234-1-lg] (Proteintech); mouse monoclonal anti-8-oxoguanine glycosylase (OGG1) [sc-376935], anti-phospho-extracellular regulated kinase (ERK) [sc-7383], mouse monoclonal anti-p53 [sc-126], rabbit polyclonal anti-cyclin A [sc-751], and anti-TOMM20 [sc-17764] (Santa Cruz); and rabbit polyclonal anti-OPA1 [NB110-55290] and anti-PINK1 [NBP2-36488] (Novus Biologicals).

### Cell survival assay

The cell survival with irradiation was measured by colony formation assay. The appropriate cells were plated on 60 mm or 100 mm tissue culture dishes before high or low dose-rate IR. After irradiation, the cells were incubated in normal cell culture conditions for 8–14 days. The cells were then fixed with 100% methanol and stained with Giemsa stain. Colonies of > 50 cells were counted. The rates of the surviving fraction were calculated using the planting efficiency (non-irradiated cells) divided by that of non-irradiated cells.

### Western blot analysis

Western blot analysis was performed as described previously [35]. Total protein (50 µg) from each whole extract was separated using sodium dodecyl sulfate-polyacrylamide gel electrophoresis and transferred to a polyvinylidene difluoride membrane, followed by blocking the membrane in 5% skim milk for 1 h at room temperature. The membrane was then incubated with primary antibodies at 4°C overnight, followed by incubation with secondary horseradish peroxidase-conjugated anti-rabbit or anti-mouse IgG (GE Healthcare) for 1 h at room temperature. Signals were then detected using an enhanced chemiluminescence detection kit.

### Immunofluorescence analysis

Immunofluorescence was performed using an Opera Phenix High Content Screening System (Perkin Elmer, Germany). Cells were cultured in DMEM and plated in 96-well plates at a proper cell number. The plates were then incubated for 24 h before radiation. The cells were fixed with 3.7% paraformaldehyde for 20 min, followed by permeabilization with 0.2% Triton X-100 for 15 min at room temperature. Next, 3% bovine serum albumin was added to the plates for 1 h at room temperature, followed by incubation with a primary antibody on a horizontal circular shaker at 70 rpm at 4°C overnight. The next day, the secondary antibodies were added to the plates for 1 h at room temperature on a horizontal shaker protected from light. Nuclei were stained using Hoechst 33342 (Thermo Scientific). Immunofluorescence images were acquired using an Opera confocal imager and were analyzed using the Opera software package.

### Measurement of total ROS

Accumulation of intercellular total ROS was analyzed by the DCFH-DA method [36]. Appropriate cells were plated on 6-well plates before high or low dose-rate irradiation. After irradiation, the cells were incubated for 0.5 h. Then 1 ml of PBS containing 50 µM 20′,70′-dichlorofluorescin diacetate (DCFH-DA) was loaded to the cells and incubated for 15 min protected from light. After incubation, the cells were observed using fluorescence microscopy with appropriate filter (OLYAMPUS-IX81; Ex/Em: 488 nm/530 nm) to acquire fluorescence images. The fluorescence images from four random areas per sample were used to measure relative fluorescence intensity by Image J software.

### Measurement of superoxide and RNS

Measurement of superoxide and RNS (reactive nitrogen species) quantity was performed using ROS/RNS Detection Kit according to the manufacturer’s instruction (Enzo Life Sciences, Farmingdale, NY, USA). Briefly, appropriate cells were plated on 6-well plates before high or low dose-rate irradiation. After irradiation, the cells were incubated for 0.5 h. Then 500 µl of ROS/RNS detection reagent was loaded to the cells and incubated in normal cell culture condition for 2 h. After incubation, the cells were observed using fluorescence microscopy with appropriate filters(OLYAMPUS-IX81; Ex/Em: superoxide: 550 nm/620 nm, RNS: 650 nm/670 nm) to acquire fluorescence images. The fluorescence images from 4–6 random areas per sample were used to measure relative fluorescence intensity by Image J software.

### Statistical analysis

The data are represented as the mean ± standard error of the mean (SEM) or standard deviation (SD). Statistical significance was analyzed using Student’s *t* test. *P* < .05 was considered significant.

## Results

### Low dose-rate IR improved cell survival compared with high dose-rate IR

**Figure 1.**
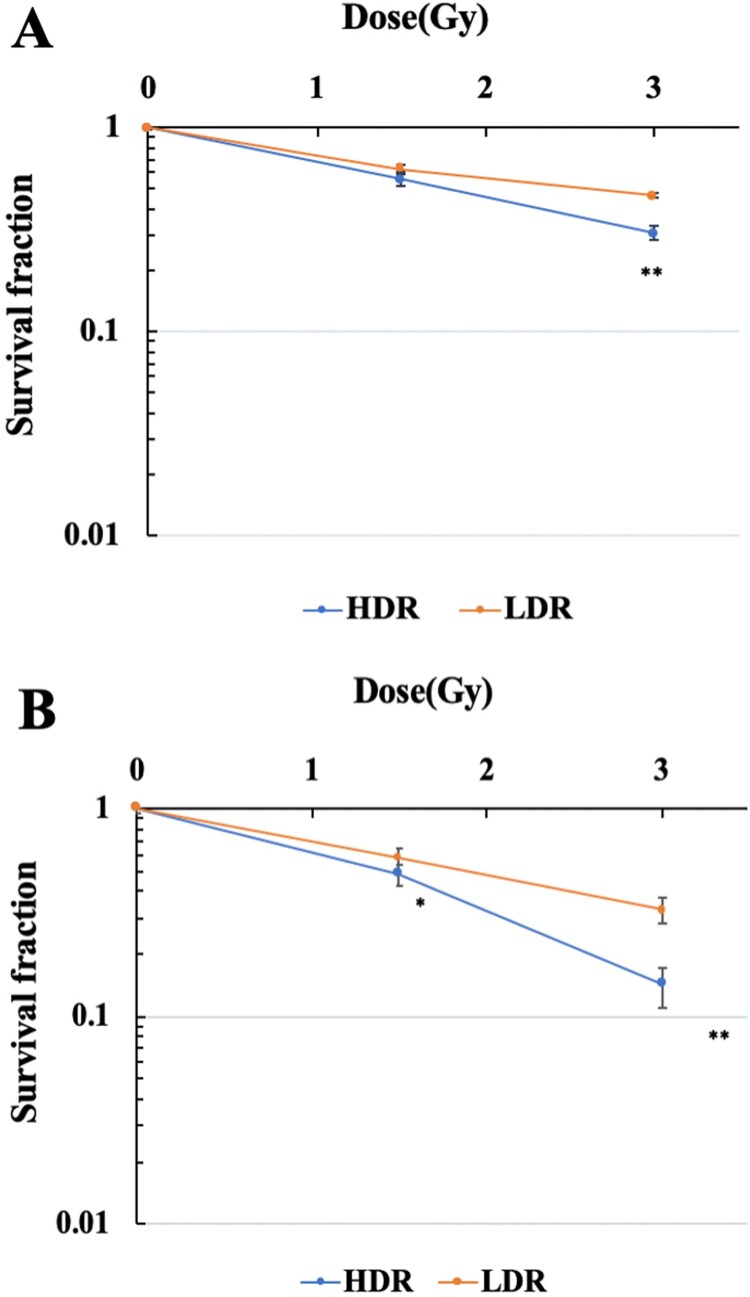
Survival curves of human normal cells and cancer cells exposed to high or low dose-rate γ-rays. 48BR (A) and HeLa (B) cells were plated to appropriate cell numbers and irradiated by high or low dose-rate γ-rays (total dose: 1.5 Gy or 3 Gy). After irradiation, the cells were incubated for 8–14 days, and the colonies were counted as shown in MATERIAL and METHODS. Data represent mean** **±** **SEM (*n* = 8), *P ≤ .05, **P ≤ .01, Student’s *t* test.

The survival curves of hTERT-immortalized human normal fibroblasts (48BR) and cancer cells (HeLa) after high (0.9 Gy/min) or low dose-rate IR (1 mGy/min) are shown in Figure 1(A and B), respectively. The viability of both 48BR and HeLa cells irradiated after low dose-rate IR increased compared with those after high dose-rate IR.

### Low dose-rate IR showed reduction of DSB damage

**Figure 2.**
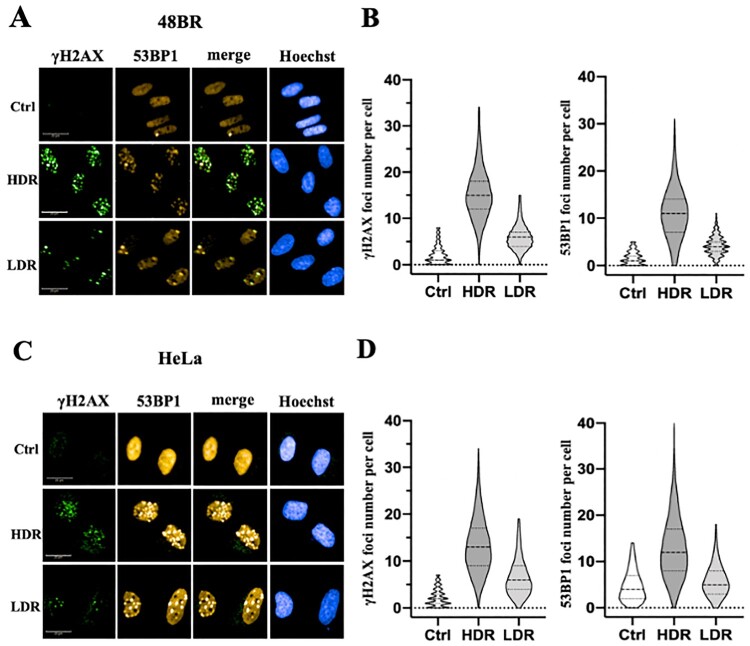
Low dose-rate IR caused less DSB damage in both normal and cancer cells. (A and B) 48BR cells and (C and D) HeLa cells were irradiated by high or low dose-rate γ-rays (total dose: 3 Gy). After incubation for 0.5 h, the cells were fixed and then immunostained with anti-53BP1 (red) and γ-H2AX (green) antibodies. The nuclei were stained by Hoechst 33342 (blue). Immunofluorescence images were acquired using Opera confocal imager. Scale bars: 20 µm. Foci were detected using the Opera confocal imager and the number of foci in a single cell was quantified using the Opera software package.

**Figure 3.**
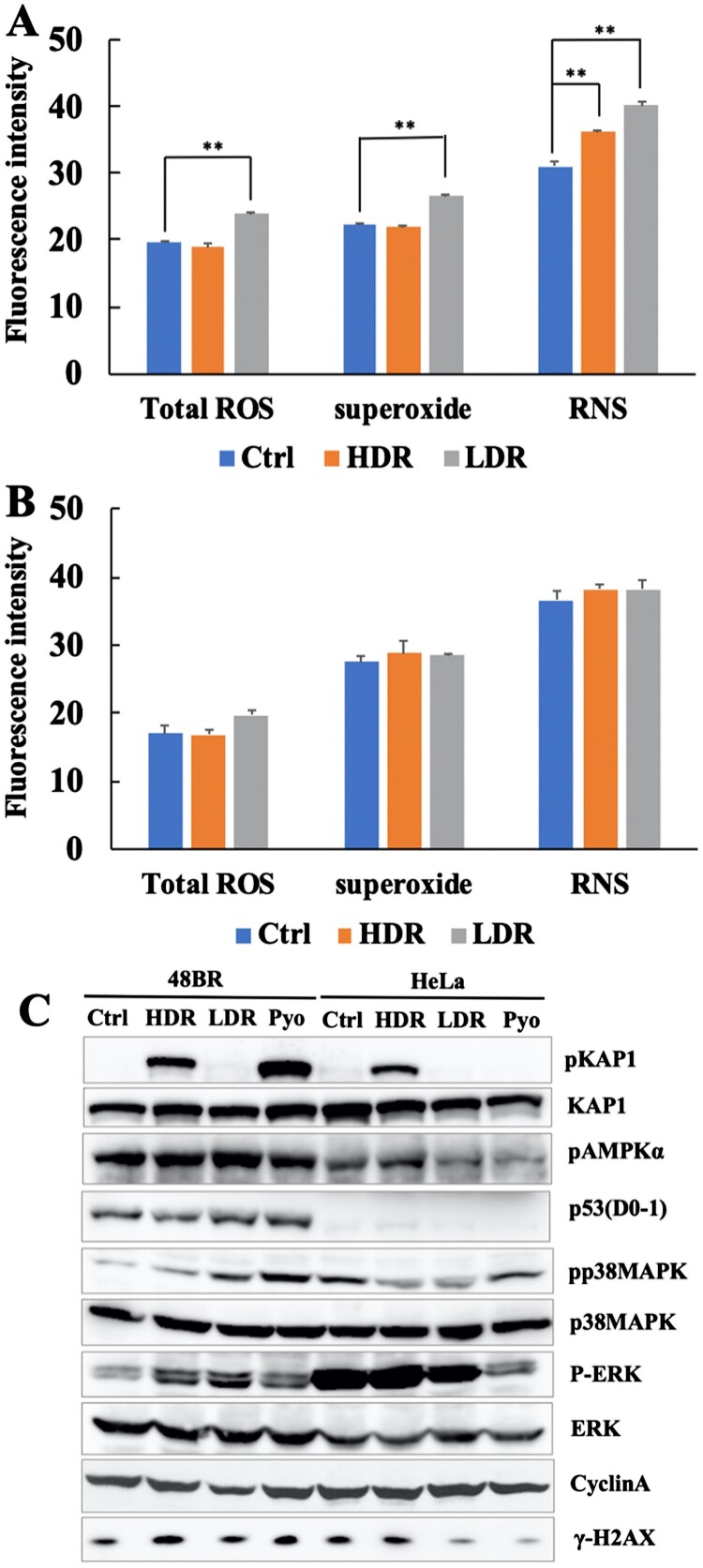
Low dose-rate IR caused increased ROS and RNS generation in human normal cells. 48BR (A) and HeLa (B) cells were irradiated by high or low dose-rate γ-rays (total dose: 3 Gy). After incubated for 0.5 h, the cells were treated with 500 μl of ROS/RNS Detection Mix for 2 h at 37°C. Then the images were captured and the intensity were analyzed using Image J software. Data represent mean ± SEM (*n* = 4–6), **P ≤ .01, Student’s *t* test. (C) Extracts from 48BR and HeLa cells, irradiated by high or low dose-rate γ-rays after incubation for 0.5 h or treated with 10 μM of pyocyanin for 2 h, as analyzed by western blot analysis using the indicated antibodies.

Exposure to high dose-rate IR immediately generates DSB damage in genomic DNA, which can be visualized with immunofluorescence using anti-γ-H2AX or 53BP1 antibodies [2]. Hence, the generation of DSB damage was investigated following low dose-rate IR in 48BR and HeLa and compared to cells receiving high dose-rate IR using these antibodies. Exposure to 3 Gy at a high dose-rate IR generated 10–20 γ-H2AX foci per nucleus in 48BR and HeLa cells, while generation of γ-H2AX foci decreased to almost 5 foci per nucleus in both 48BR (Figure 2(A and B)) and HeLa cells (Figure 2(C and D)) following low dose-rate IR. Focus formation of 53BP1, a factor involved in the DSB damage-dependent pathway, was also examined, and a similar tendency as γ-H2AX was observed (Figure 2(A–D)). High dose-rate IR induced approximately 10 foci of 53BP1 in both 48BR and HeLa cells, while low dose-rate IR caused approximately 5 foci. Furthermore, Figure 2(A and C) shows colocalization between the γ-H2AX and 53BP1 foci under both the low dose-rate IR and the high dose-rate IR in 48BR and HeLa cells. Examination of γ-H2AX by western blot analysis also showed a similar tendency under low dose-rate IR in both 48BR and HeLa cells (Figure 3(C)). In response to DSB damage, ATM kinase is activated and then phosphorylates several substrates, including KAP1, to repair DSB damage [2, 22]. Western blot analysis of 48BR and HeLa cells showed that high dose-rate IR induced KAP1 phosphorylation, whereas low dose-rate IR did not (Figure 3(C)). These results indicated that low dose-rate IR caused a lower frequency of DSB damage than that in cells receiving high dose-rate IR.

### Low dose-rate IR caused increased ROS and activation of the oxidative stress response

As high dose-rate IR is known to increase ROS and RNS and subsequent activation of the oxidative stress response [3, 4], ROS and RNS were investigated after exposure to low dose-rate IR as described in MATERIALS AND METHODS (Figure 3(A and B) and Figure S1(A and B)). For 48BR cells, low dose-rate IR increased total ROS, as well as superoxide, mainly generated in mitochondria, compared to that in cells receiving high dose-rate IR or non-irradiated cells (Ctrl). However, the RNS increased after both high and low dose-rate IR. In contrast, HeLa cells did not show remarkable increases in both total ROS, superoxide and RNS under both low and high dose-rate IR compared to non-irradiated cells.

To investigate whether the oxidative stress response was activated following ROS accumulation, several factors involved in this response were examined using western blot analysis in irradiated and pyocyanin (ROS inducer)-treated cells. One of the famous tumor suppressor gene products, p53, is known to respond to both DNA damage and increased ROS [37]. As shown in Figure 3(C), low and high dose-rate IR, as well as pyocyanin treatment, caused elevated levels of p53 in 48BR cells, but not in HeLa cells. AMPK, ERK, and p38MAPK are activated by phosphorylation via ROS [38, 39]. In 48BR cells, phosphorylation of AMPK, p38MAPK, and ERK was observed after both irradiation and pyocyanin treatment. Notably, phosphorylation levels of p38MAPK and ERK under low dose-rate IR were higher than those under high dose-rate IR. However, for HeLa cells, both irradiation and pyocyanin treatment did not induce increased phosphorylation of AMPK, p38MAPK, and ERK (Figure 3(C)). OGG1 is a major factor involved in base excision repair, which is responsible for the repair of oxidative DNA damage [40]. In 48BR cells, high and low dose-rate IR, as well as pyocyanin treatment, caused increased expression of OGG1, whereas HeLa cells did not express OGG1 in any situation (Figure 3(C)). These results indicated that low dose-rate IR induced activation of the oxidative stress response in normal cells, but not in cancer cells.

Cell cycle distribution was analyzed using the Opera Phenix High Content Screening System and it was found that low dose-rate IR increased numbers of G1 phase cells from 0.5 h post-irradiation in 48BR cells, while high dose-rate IR did not (Figure S2(A)). For HeLa cells, there were no remarkable changes observed in the cell cycle distribution after both low and high dose-rate IR (Figure S2(B)). Cyclin A, which is expressed in the late G1 phase, contributes to the transition to the G2 phase and reaches its peak in G2 phase [41]. In agreement with the cell cycle assay, western blot analysis showed reduced levels of cyclin A after low dose-rate IR, but no change after high dose-rate IR compared to that in non-irradiated 48BR cells. In HeLa cells, cyclin A levels remained unchanged in both high and low dose-rate irradiated HeLa cells (Figure 3(C)). These results suggested that 48BR cells arrest in the G1 phase after low dose-rate IR. Furthermore, the data in Figure 3(C) suggest the activation of the oxidative stress response only in normal 48BR cells; therefore, oxidative stress responses, biological effects, and molecular mechanisms under low dose-rate IR were further investigated in normal 48BR cells.

### Low dose-rate IR induced mitochondrial changes

**Figure 4.**
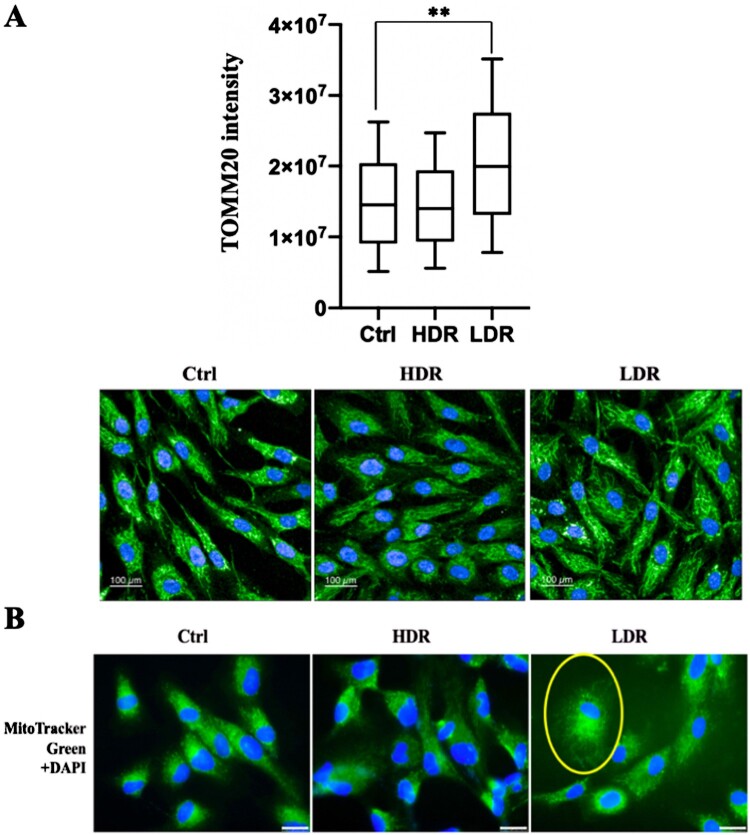
Mitochondrial mass increase and mitochondrial morphological change after low dose-rate IR. (A) Immunofluorescence images of 48BR cells irradiated by high or low dose-rate γ-rays and harvested after 0.5 h (total dose: 3 Gy). The cells were fixed and immunostained with anti-TOMM20 antibody. Immunofluorescence images (lower panel) were acquired using an Opera confocal imager and the intensity of TOMM20 (upper panel), as an indicator of mitochondrial mass, and the mean, SD and *p*-value (**P ≤ .01, Student’s *t* test) was also calculated using the Opera software package. Scale bars: 100 µm. (B) Fluorescence images of 48BR cells irradiated by high or low dose-rate γ-rays (total dose: 1.5 Gy) after incubation for 0.5 h. Cells were stained using Mito Tracker Green (MitoTracker^®^ Mitochondrion-Selective Probes, Invitrogen) to visualize mitochondria. Nuclei were stained for fluorescence using Vectashield^®^ Mounting Medium with 4′,6-diamidino-2-phenylindole (DAPI, Vector Laboratories, Inc.). Scale bars: 25 µm.

Mitochondria is the major generator of ROS in cells, and a high amount of ROS is associated with mitochondrial changes related to mitochondrial damage under stress conditions [8–10]. Additionally, high dose-rate IR induces various mitochondrial changes, some of which are associated with ROS accumulation [6, 18, 19]. Hence, whether low dose-rate IR caused mitochondrial changes were investigated. First, mitochondria were stained by immunofluorescence using an anti-TOMM20 (mitochondrial outer membrane protein) antibody (Figure 4(A), lower panel). The quantitative analysis performed by the Opera Phenix High Content Screening System showed an increase (almost two-fold) in TOMM20 expression after low dose-rate IR compared to that in non-irradiated cells, and a slight decrease in cells receiving high dose-rate IR (Figure 4(A), upper panel). Mitochondria were stained with Mito Tracker Green (Figure 4(B)) and induced changes in mitochondrial distribution were observed after low dose-rate IR, whereas no change was observed in cells receiving high dose-rate IR or non-irradiated cells. Changes in mitochondrial morphology or mass are accompanied by mitochondrial defects, which is related with the MMP [42]. The MMP was examined after irradiation using the MMP detection kit (JC-10) and the MMP geometric mean was elevated at 0.5 and 24 h after low dose-rate IR, whereas the MMP was nearly unchanged after high dose-rate IR (Figure S3). Taken together, low dose-rate IR induced mitochondrial changes, accompanied with ROS increases.

### Reduction of mitophagy-related factors induced by low dose-rate IR contributed to oxidative stress

**Figure 5.**
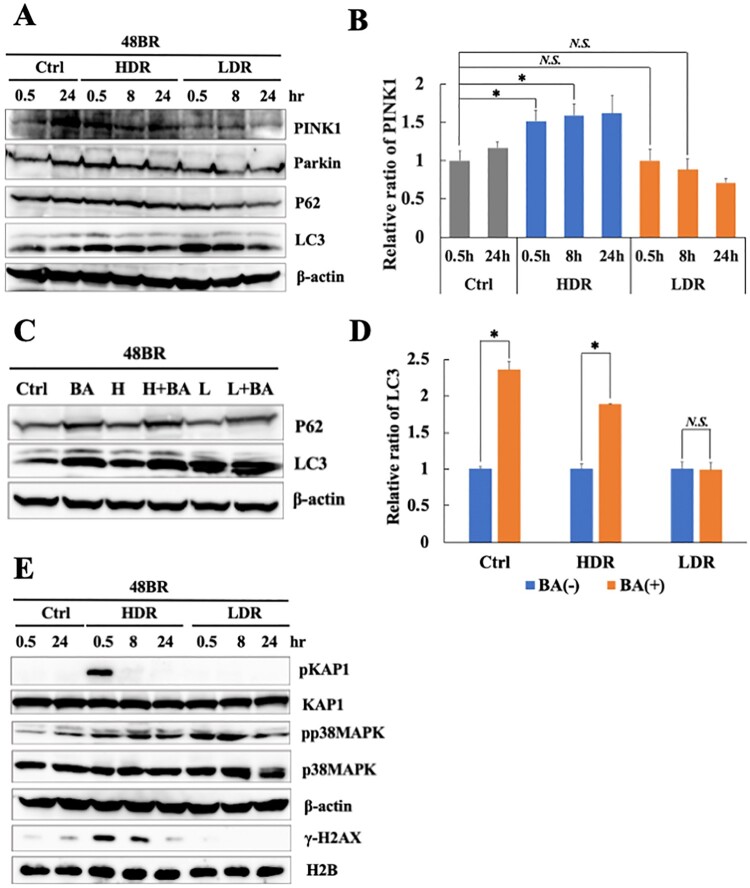
Mitophagy-related factor reduction and continuous activation of the oxidative stress response following low dose-rate IR. (A and E) Extracts from 48BR cells, irradiated with high or low dose-rate γ-rays (total dose: 3 Gy) and incubated at the indicated time points post-irradiation, as analyzed by western blot analysis using the indicated antibodies. (B) Data of PINK1 represent mean ± SEM (*n* = 3; containing (A)), *P ≤ .05, N.S means no significant difference, Student’s *t* test. (C) Extracts from 48BR cells, as analyzed by western blot analysis with the indicated antibodies. 48BR cells, including high dose rate and non-irradiated cells were pretreated with Bafilomycin A (BA, 100 nM) immediately before low dose-rate γ-ray irradiation (total dose: 3 Gy) and incubated for 0.5 h after irradiation. (D) Data of LC3 represent mean ± SEM from. (*n* = 3; containing (C)), *P ≤ .05, N.S means no significant difference, Student’s t test.

As shown in Figure 4 and Figure S3, low dose-rate IR induces several mitochondrial changes, which are associated with mitochondrial damage. Mitophagy specifically removes damaged mitochondria, which are responsible for excess ROS accumulation in a cell [11–13]. As the relationship between IR-induced mitochondrial changes and mitophagy was unknown, mitophagy-related factors were investigated under low dose-rate IR using western blot analysis. As shown in Figure 5(A), p62 were unchanged in irradiated cells compared to that in non-irradiated cells. LC3 increased at 0.5 h and 8 h both in high and low dose-rate IR. However, the key factor, PINK1, significantly increased until 8 h after high dose-rate IR while was unchanged after low dose-rate IR compared to that in non-irradiated cells (Figure 5(A and B)), Parkin also increased slightly at 0.5 h after high dose-rate IR while was unchanged after low dose-rate IR. These results suggested that high dose-rate IR stimulated PINK1-dependent mitophagy, whereas low dose-rate IR might not.

BA, which is known to inhibit autophagy, inhibits the fusion of autophagosomes with lysosomes [34]. Additionally, BA also inhibits mitophagy [43]. Hence, the activation of mitophagy was examined after IR and pretreatment with BA. BA pretreatment increased levels of p62 and LC3 in non-irradiated cells (Figure 5(C and D)), indicating that this treatment sufficiently inhibited the mitophagy pathway. In high dose-rate irradiated cells, BA pretreatment caused LC3 accumulation significantly, suggesting that high dose-rate IR induced mitophagy activation. In the case of low dose-rate IR, LC3 did not increase with BA treatment (Figure 5(C and D)), suggesting that mitophagy might be inactive under low dose-rate IR.

Mitophagy plays an important role in preventing ROS accumulation by removing damaged mitochondria. Hence, the continuation of the oxidative stress response after IR was investigated using western blot analysis. As shown in Figure 5(E), increased levels of p38 MAPK phosphorylation are observed until 8 h after low dose-rate IR, suggesting the continuous activation of the oxidative stress response following low dose-rate IR. In contrast, KAP1 phosphorylation in response to DSB damage following high dose-rate IR disappeared after 24 h. The results are shown in Figure 5 suggest that the continuation of the activation of the oxidative stress response following low dose-rate IR is related to mitophagy repression.

### Decreased mitochondrial fusion events were responsible for PINK1-dependent mitophagy repression under low dose-rate IR

**Figure 6.**
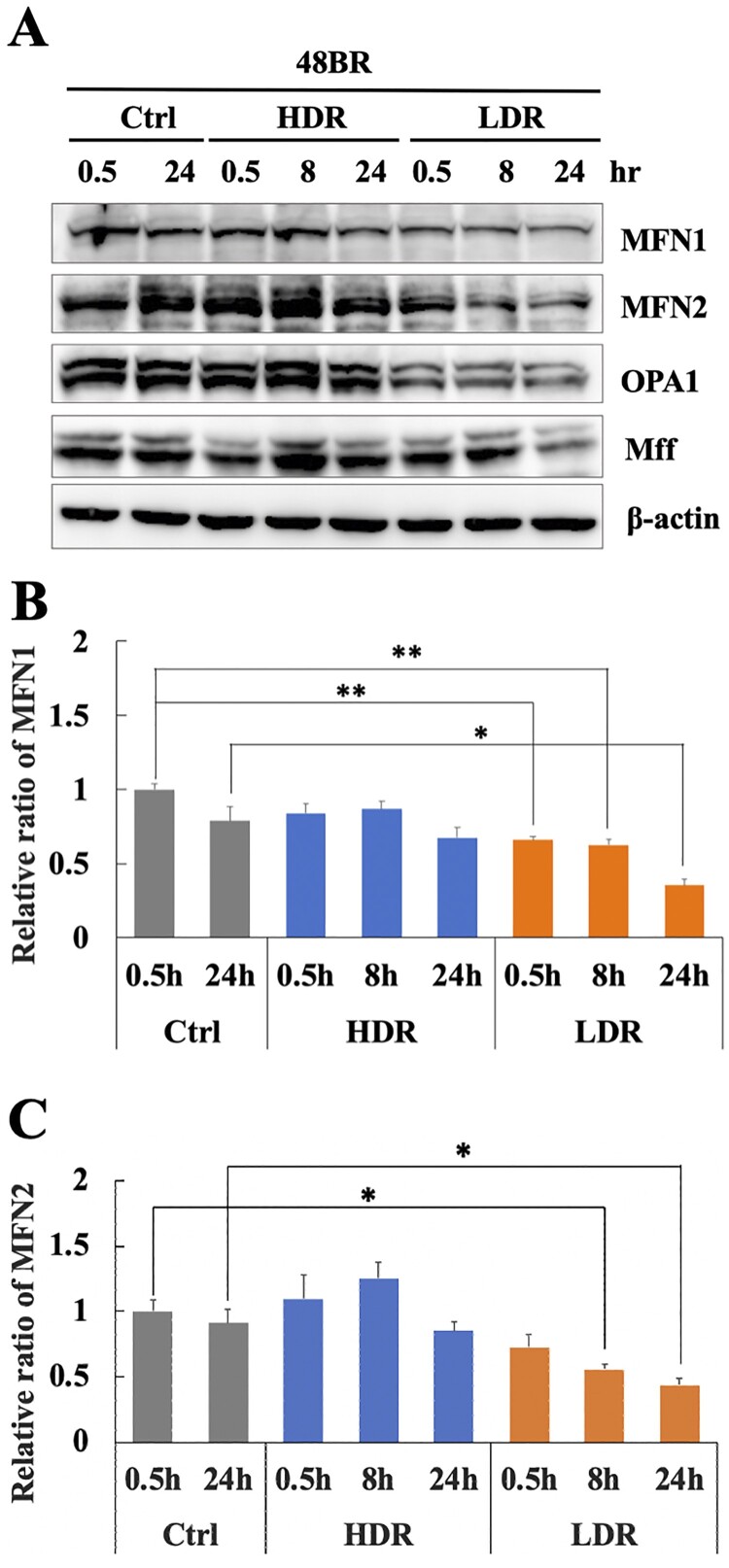
Low dose-rate IR induces a reduction in mitochondrial fusion regulators. (A) Extracts from 48BR cells, irradiated by high or low dose-rate γ-rays (total dose: 3 Gy) and incubated at the indicated time points post-irradiation, as analyzed by western blot analysis using the indicated antibodies. Data of MFN1 (B) or MFN2 (C) represent mean ± SEM (*n* = 3; containing (A)), *P ≤ .05, N.S means no significant difference, Student’s t test.

Mitochondrial fission and fusion events play important roles in the removal of damaged mitochondria and the integration of undamaged mitochondria into the mitochondrial network during mitophagy [16, 17]. Therefore, mitochondrial fission/fusion-related factors were investigated under low dose-rate IR using western blot analysis. Figure 6 shows that the mitochondrial fusion factors, MFN1 and MFN2, significantly decreased under low dose-rate IR compared to that in non-irradiated cells. However, these factors were unchanged after high dose-rate IR. Another fusion factor, OPA1, also decreased after low dose-rate IR (Figure 6(A)), while a fission factor, MFF changed slightly with irradiation. These results suggested that decreased mitochondrial fusion factors following low dose-rate IR was related to the inhibition of PINK1-dependent mitophagy activation.

## Discussion

**Figure 7.**
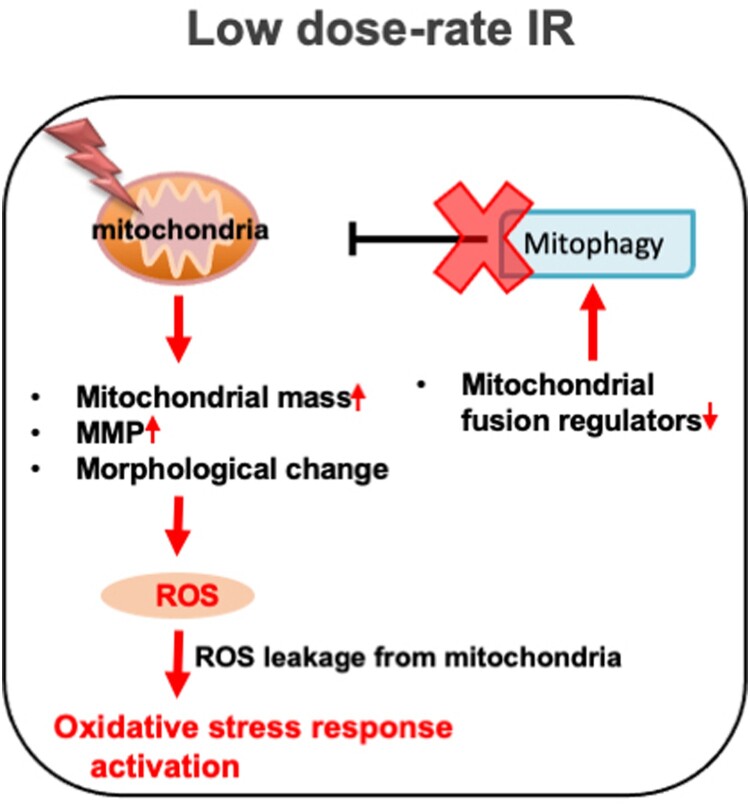
Low dose-rate IR causes ROS accumulation and activation of oxidative stress responses via repression of mitophagy. Exposure to low dose-rate IR reduced mitochondrial fusion regulators, leading to repression of mitophagy. The repression of mitophagy accumulates damaged mitochondria, which causes excess ROS accumulation and subsequent activation of oxidative stress responses.

Chronic oxidative stress evoked by continuous ROS accumulation induces genomic instability, which is highly correlated with carcinogenesis and several degeneration disorders [26–28]. High dose-rate IR induces ROS accumulation and subsequent oxidative stress, while there is no clear information on biological effects under low dose-rate IR. Hence, the molecular mechanism involved in ROS generation in normal human cells was clarified. Remarkable increases in ROS levels (Figure 3(A)) and activation of the oxidative stress response were observed following low dose-rate IR in human normal 48BR cells (Figure 3(C)). Several mitochondrial changes were also observed after low dose-rate IR, including morphology, mass and MMP, which were related to mitochondrial dysfunction (damage) (Figure 4 and Figure S3). Furthermore, the mitophagy-related factor PINK1 was increased in cells following high dose-rate IR, but not low dose-rate IR (Figure 5(A and B)). Moreover, decreased levels of mitochondrial fusion regulators following low dose-rate IR were observed (Figure 6). Taken together, the reduction of mitochondrial fusion regulators under low dose-rate IR leads to repression of mitophagy and accumulation of damaged mitochondria by IR (Figure 7). The damaged mitochondria leak excess ROS and subsequent activation of oxidative stress responses.

A remarkable mitochondrial mass increase was observed after low dose-rate IR (Figure 4(A)). All the mitochondrial fusion regulators showed decreased expression until 24 h after low dose-rate IR (Figure 6). Under physiological conditions, the mitochondrial fission/fusion cycle is sequential and cyclic (about 20 min for one cycle in neurons) [44]. Thus, the decrease in fusion regulators might cause excessive division of mitochondria, which might be related to the increased mass. However, the balance between mitochondrial fission and fusion events plays an important role in the execution of mitophagy [16, 17]. Since mitophagy plays a key role in reducing mitochondrial mass in response to stresses [45–47], the imbalance between mitochondrial fission and fusion events may also contribute to mitochondrial mass increase by disturbing the mitophagy process under low dose-rate IR.

Pre-treatment with an ATM inhibitor repressed an increase of the mitophagy factor PINK1 in response to high dose-rate IR (Figure S4). Mitochondrial damage inducer (carbonyl cyanide m-chlorophenylhydrazone) treatment, which induces mitophagy, does not increase mitophagy-regulator proteins in the mitochondrial fraction of ATM-null cells [48]. Additionally, the translocation of Parkin to mitochondria and the subsequent colocalization with LC3 in the spermidine-induced mitophagy pathway are disrupted by pre-treatment with an ATM-specific inhibitor [49]. ATM could play an important role in maintaining mitophagy activation, and might also participate in mitophagy caused by high dose-rate IR.

Significant increases in cancer risk for leukemia and solid cancers, such as breast cancer, under chronic IR at low dose rates have been reported [50–53]. Oxidative stress has been linked to various pathologies, including cancer [54, 55]. As the generation of DSB damage is too low under such conditions, oxidative stress evoked by excess ROS accumulation may significantly contribute to tumorigenesis. Oxidative stress is caused by an imbalance between ROS generation and the capacity to remove these ROS [56]. In addition to the ROS elimination process (mitophagy), anti-oxidative pathways, such as anti-oxidative enzymes and their regulatory systems, are also very important in regulating ROS levels in a cell [57]. Hence, further studies on the capacity of complete anti-oxidative pathways are important to clarify how chronic IR at low dose rates may or may not lead to tumorigenesis.

## Supplementary Material

Supplemental Material
