# Supplementary material for: Possible relationship between mitochondrial changes and oxidative stress under low dose-rate irradiation
Source: Redox Rep. 2021 Aug 26;26(1):160–9. doi: 10.1080/13510002.2021.1971363 (PMC8405122; doi:10.1080/13510002.2021.1971363)
Supplement: Supplemental Material [file YRER_A_1971363_SM0540.docx]

**A**

**
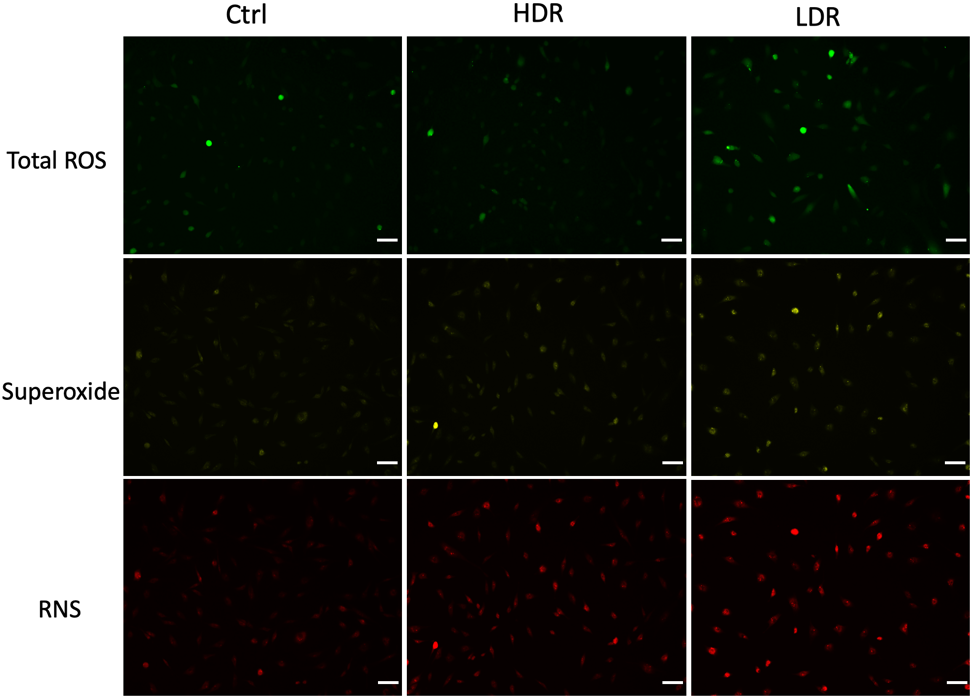
**

**B**

**
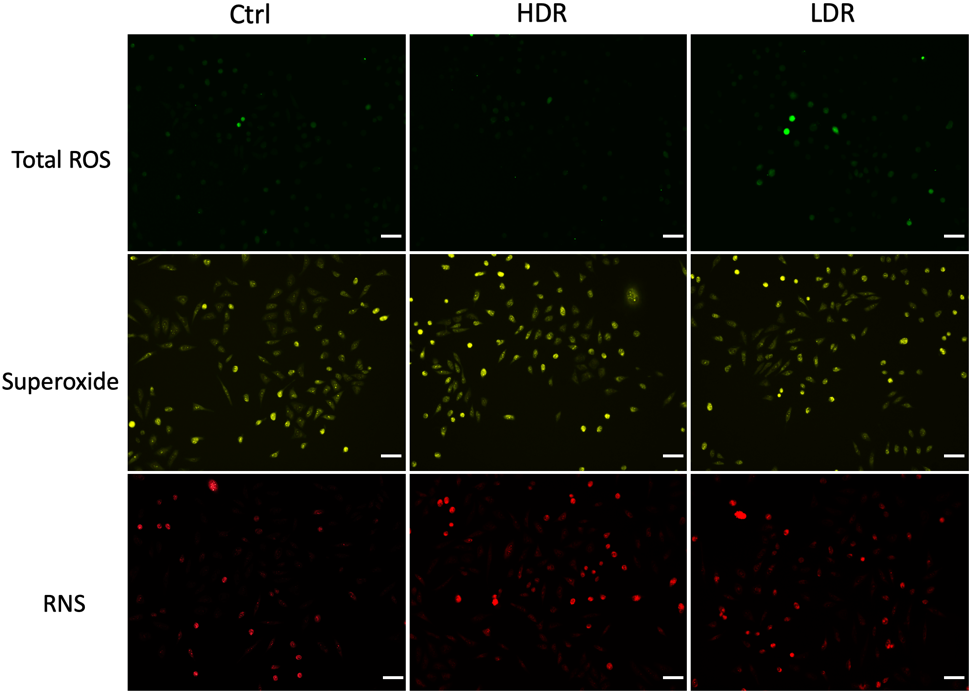
**

**Fig. S1. Fluorescence images of 48BR cells and HeLa cells depicting ROS and RNS generation following high or low dose-rate IR**

(A) 48BR and (B) HeLa cells were irradiated by high or low dose rate γ-rays (total dose: 3 Gy). After incubated for 0.5 h, the cells were treated with 500 μl of ROS/RNS Detection Mix for 2 h or 1 ml of DCFH-DA for 15 min at 37°C. Then the Fluorescence images were captured. Scale bars: 100 µm.

**
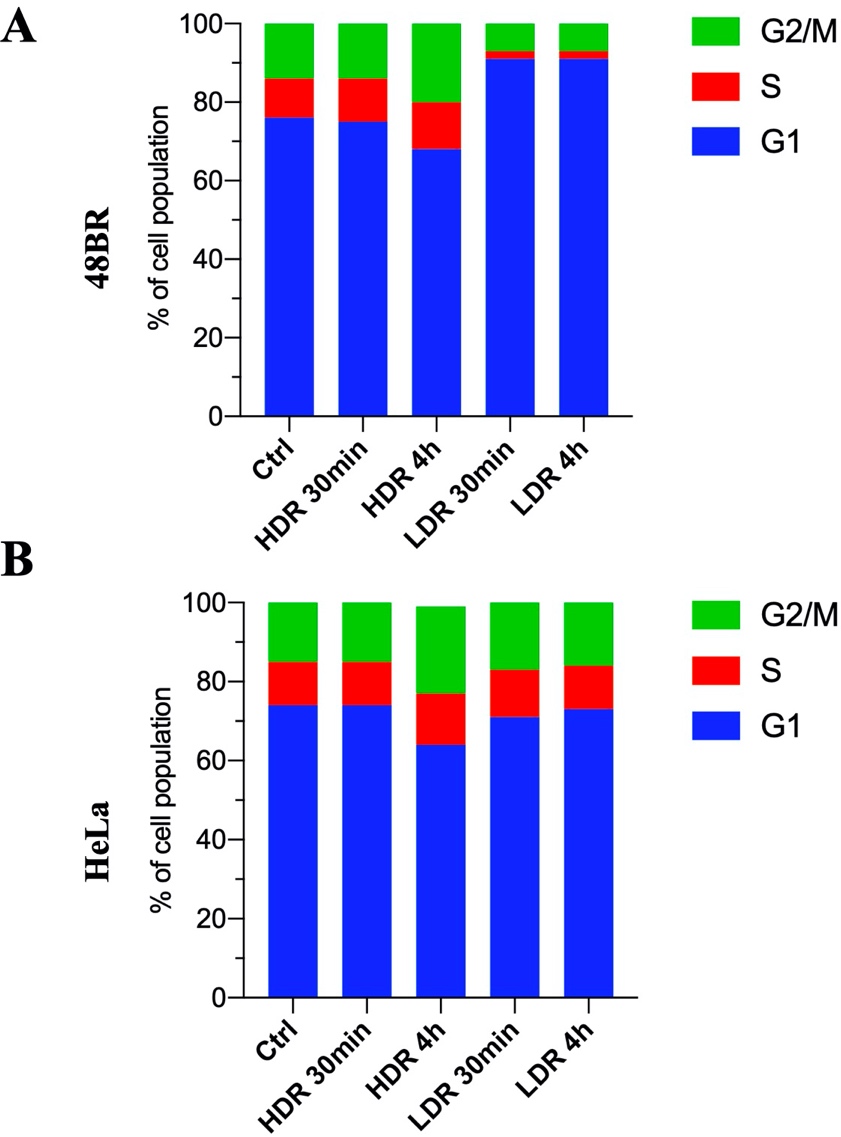
**

**Fig. S2. Low dose rate IR induced G1 phase arrest in human normal cells**

(A) 48BR cells and (B) HeLa cells were irradiated by high or low dose rate γ-rays (total dose: 3 Gy). After incubated for 0.5 h and 4 h, the nuclei were stained by Hoechst 33342, and cell cycle was analyzed by quantifying the intensity of Hoechst 33342 using Opera software package.


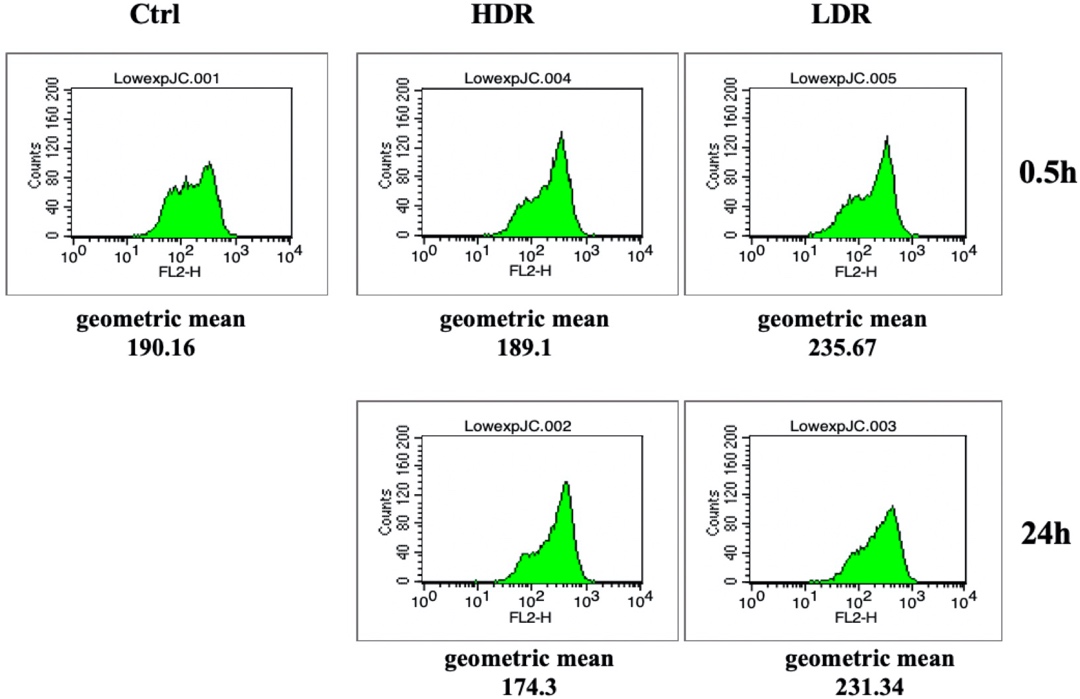


**Fig. S3. Increased MMP level caused by low dose-rate IR**

48BR cells were irradiated by high or low dose rate γ-rays (total dose: 3 Gy). After incubation for 0.5 h and 24 h, the cells were then stained using Cell Meter JC-10 Mitochondrial Membrane Potential Assay Kit (AAT Bioquest), under protective condition from light for 0.5 h. The fluorescence intensity was monitored using FACSCalibur^TM^ flow cytometer (Becton Dickinson).

**
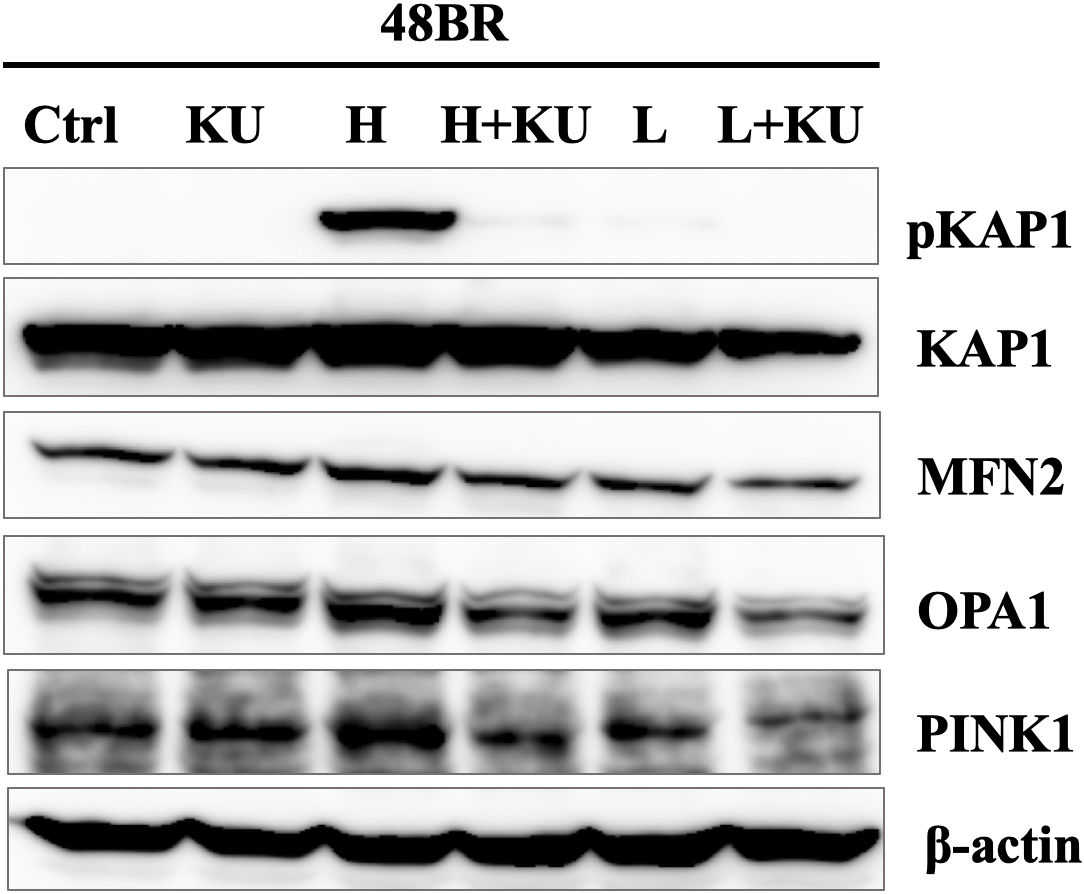
**

**Fig. S4. The effect of ATM inhibition to mitochondria-related factors**

Extracts from 48BR cells, was analyzed by western blot analysis with the indicated antibodies. 48BR cells, including high dose-rate and non-irradiated cells were pretreated with KU55933 (10 μM) immediately before low dose-rate γ-ray irradiation (total dose: 3 Gy) and incubated for 0.5 h after irradiation.


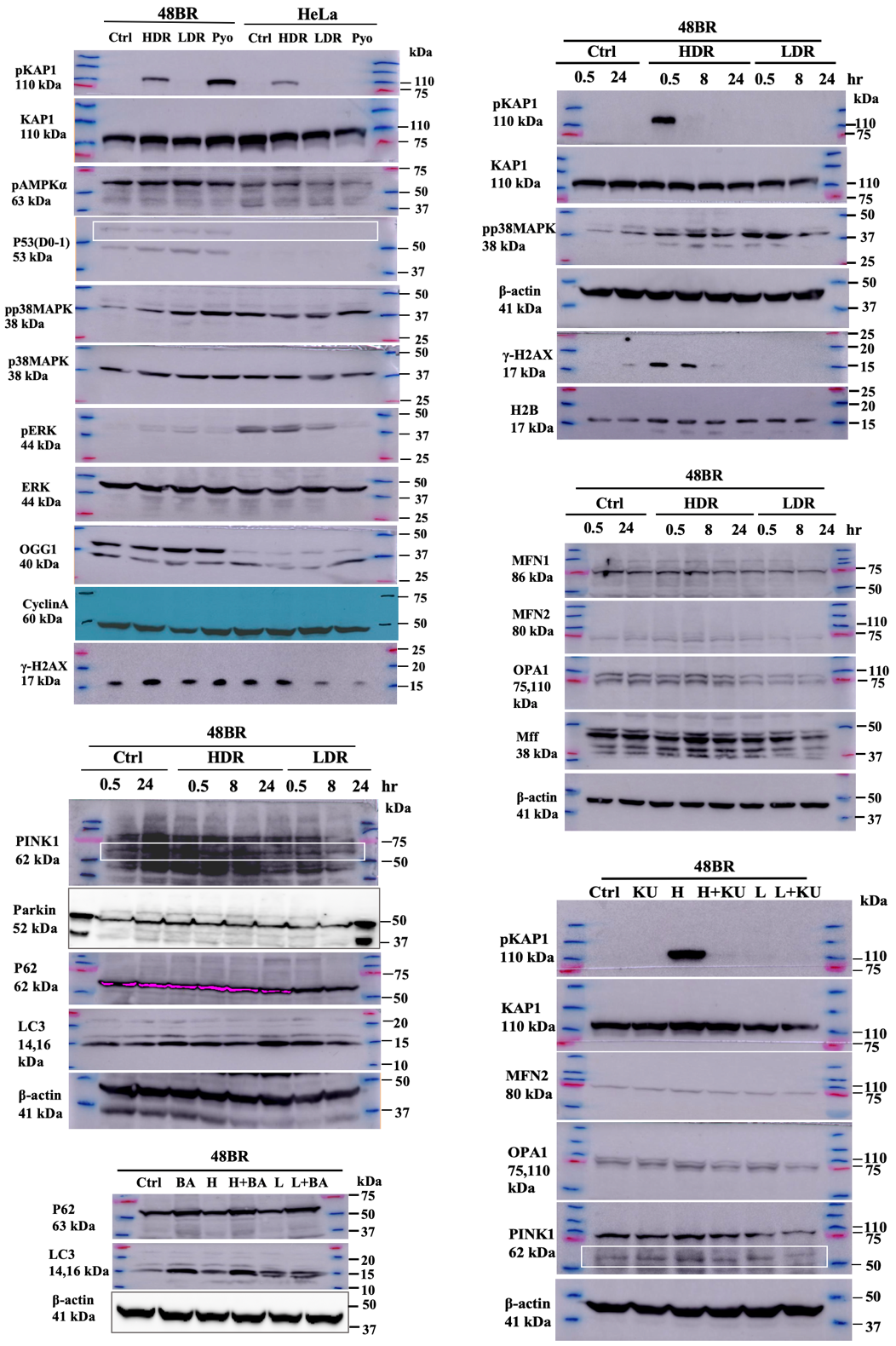


**F**

**E**

**C**

**B**

**A**

**D**

**Figure. S5. Western blot data with molecular weight marker.**

(A) for Fig. 3. (B) for Fig. 5A. (C) for Fig. 5C. (D) for Fig. 5E (E) for Fig. 6A. (F) Fig. S4.
